# A Fœtus Arrested in Its Devolopment at the Fourth Month, and Carried to the Full Period of Gestation; or, Two Hundred and Seventy-Six Days

**Published:** 1877-05

**Authors:** E. Day

**Affiliations:** Grand Tower, Ill.


### A FŒTUS ARRESTED IN ITS DEVELOPMENT AT THE FOURTH MONTH, AND CARRIED TO THE FULL PERIOD OF GESTATION; OR, TWO HUNDRED AND SEVENTY-SIX DAYS.

By E. DAY, M. D., Grand Tower, Ill.

The history of the following case is presented to the medical profession, not because it is supposed to possess much practical importance, but as showing what does sometimes happen under certain conditions and circumstances.

On the 8th of August last, I was consulted, at my office, by Mrs. I. M., aged 26 years, married, and residing about three miles from Grand Tower, in regard to her condition; she believing herself to be in the eighth month of pregnancy. Her appearance, and the history of her case, gave strong assurance of the fact.

She made the following statement: Her catamenia ceased on the 5th of December, 1875, (with one exception hereafter mentioned) after which a gradual enlargement of her abdomen took place, as in her previous pregnancy, and continued for five or six months; but, of late, this development has not kept pace with her advancing gestation.

Her health had been, and still continued good. Her mind, however, has been much disquieted, from the fact that she has never felt any movement, indicating fœtal life, as she experienced during her first pregnancy; and, also, from the fact that a recurrence of the catamenia took place about nine days since, and lasted three days.

She appeared to be in an advanced stage of gestation.

Under these circumstances, I did not find it necessary to ascertain by the aid of the stethoscope whether the *bruit placentaire* existed; or to remove any lingering doubts in regard to the correctness of my diagnosis.

I dismissed the case, as one not requiring any medical interference, and advised her to banish all anxiety from her mind, as there appeared nothing in her case of an alarming character; and assuring her that a few weeks would perhaps demonstrate that, "whatever is, is right."

On the morning of September 6th, 1876, I was summoned to attend her at her confinement.

I was informed, on my arrival, that she had been in labor several hours; and, on examination, found the os uteri well dilated with the amniotic bag protruding. A few pains sufficed to complete the work of expelling, unruptured, the membranous sac, with its contents, and also the placenta.

In the membranes there were enclosed *the remains of a fœtus, of about four months development*, but really nine months of age.

The sac contained also about four ounces of thickened amniotic fluid, free from any offensive odor. The fœtus measured eight inches in length, when straightened; and weighed a little more than nine ounces. The tissues of the fœtus had been absorbed to such an extent, as to leave only the integument and hardened muscles attached to the skeleton; the placenta also had undergone a similar process of absorption.

As the uterus had been tolerant of a fœtus, arrested in its development and slowly undergoing absorption, for four or five months, may it not be rational to infer that the mental condition of this patient—believing that she had reached the full period of her pregnancy—had the effect of inducing labor pains and the expulsion of the contents of the uterus; which otherwise might have been retained to an indefinite period?

As I did not subject my patient to an examination, in the first instance, and without giving much importance to the wanting link in the chain of symptoms, of normal gestation, I concluded, from the absence of the catamenia, for seven or eight months, the enlargement of the mammary glands, and the general embonpoint of my patient, that she was proceeding to the full period of a natural pregnancy; and consequently was not a little surprised at the singular denouement of the case.

